# Socioeconomic and geographic differences in ablation of atrial fibrillation in Norway - a national cohort study

**DOI:** 10.1186/s12889-022-12628-9

**Published:** 2022-02-14

**Authors:** Frank Olsen, Bård Uleberg, Bjarne K. Jacobsen, Ivar Heuch, Pål M. Tande, Einar Bugge, Lise Balteskard

**Affiliations:** 1 Department of Community Medicine, UiT The Arctic University of Norway, Tromsø, Norway; 2 Centre for Clinical Documentation and Evaluation (SKDE), Northern Norway Regional Health Authority, Tromsø, Norway; 3 Centre for Sami Health Research, UiT The Arctic University of Norway, Tromsø, Norway; 4 Department of Mathematics, University of Bergen, Bergen, Norway; 5 Department of Cardiology, University Hospital of North Norway, Tromsø, Norway; 6 Department of Clinical Medicine, UiT The Arctic University of Norway, Tromsø, Norway; 7 Centre for Clinical Research and Education, University Hospital of North Norway, Tromsø, Norway

**Keywords:** Norway, Atrial fibrillation, Catheter ablation, Universal health care, Socioeconomic factors, Small-area analysis

## Abstract

**Background:**

The aim of this study was to analyse whether there are patient related or geographic differences in the use of catheter ablation among atrial fibrillation patients in Norway.

**Methods:**

National population-based data on individual level of all Norwegians aged 25 to 75 diagnosed with atrial fibrillation from 2008 to 2017 were used to study the proportion treated with catheter ablation. Survival analysis, by Cox regression with attained age as time scale, separately by gender, was applied to examine the associations between ablation probability and educational level, income level, place of residence, and follow-up time.

**Results:**

Substantial socioeconomic and geographic variation was documented. Atrial fibrillation patients with high level of education and high income were more frequently treated with ablation, and the education effect increased with increasing age. Patients living in the referral area of St. Olavs Hospital Trust had around three times as high ablation rates as patients living in the referral area of Finnmark Hospital Trust.

**Conclusions:**

Differences in health literacy, patient preference and demands are probably important causes of socioeconomic variation, and studies on how socioeconomic status influences the choice of treatment are warranted. Some of the geographic variation may reflect differences in ablation capacity. However, geographic variation related to differences in clinical practice and provider preferences implies a need for clearer guidelines, both at the specialist level and at the referring level.

**Supplementary Information:**

The online version contains supplementary material available at (10.1186/s12889-022-12628-9).

## Background

Atrial fibrillation (AF) is the most common cardiac arrhythmia, with significant influence on quality of life, morbidity and mortality [1–6]. The prevalence of AF has been increasing over the last decades, and is expected to increase further over the next 30 to 50 years [2, 7–10]. Thus, AF has become an important public health issue and a significant contributor to health care cost in the Western world.

Over the last two decades, catheter ablation has evolved as an important treatment option for many patients with symptomatic AF, with reasonable success rates, low complication rates and acceptable cost-effectiveness [3, 5, 11]. The procedure was primarily indicated for patients without structural heart disease, where rhythm control is the strategy of choice and in whom medical therapy has failed [4]. However, more recently, catheter ablation has also increasingly been considered as first-line therapy in selected individuals [3, 6, 12, 13].

In 2010 the Norwegian Ministry of Health and Care Services instructed the regional health authorities (RHA) to increase the capacity for catheter ablation of AF, as there was an increasing discrepancy between demand and capacity for catheter ablation in Norway. This led to a substantial increase in the number of radiofrequency ablation procedures performed within the national health care system. By 2013, Norway was near the top in Europe in number of AF ablations performed per million inhabitants [14].

In Norway, only five hospitals are performing AF ablations, one in each of the four RHAs. In addition, one private hospital in the South-East RHA is performing the procedure as a subcontractor for the regional health authority.

Norway has a universal health care system and in-hospital treatment is free of charge. It is a fundamental principle in this system that equal needs should be met by equal services regardless of e.g., socioeconomic status (SES) or place of residence. However, an increasing number of studies indicate that this principle is not adequately met, in Norway as in other Western countries [15–19]. Several studies report socioeconomic differences in utilisation of health care, e.g. relatively wealthy and/or highly educated people visit more specialists and have more access to sophisticated therapies [16–19]. Furthermore, several decades ago Wennberg reported on small area variations in health care delivery, which could not be explained by corresponding variations in need [20]. Geographic variation in access to health care in Norway has been documented in a broad spectrum of services [15, 21, 22], especially by the Norwegian health care Atlases [23].

According to the equity aims of the Norwegian health care system, treatment with catheter ablation of AF should be distributed according to disease prevalence regardless of socioeconomic class and place of residence. The aim of the present study was to analyse whether there are patient related or geographic differences in the use of this procedure among patients diagnosed with AF.

## Methods

### Study design and data sources

The study population was the complete cohort of all Norwegians, aged 25 to 75, diagnosed with atrial fibrillation by Norwegian hospitals/specialist health care providers, in Norway in the period 1 January 2008 to 31 December 2017. Data from the Norwegian Patient Register (NPR) and Statistics Norway (SSB) were linked by an encrypted serial number derived from the unique 11-digit personal identifier held by all persons living in Norway. The data from NPR included patient demographics (residential information, year of birth and gender), start and end date for the contact, hospital, type of contact, diagnoses and clinical procedures. In Norway, all hospitals submit data to NPR for registration and reimbursement purposes. The data from SSB included income and level of education each year, gender, year of birth, date of death, date of emigration and residential municipality.

### Definitions

The data were analysed by survival analysis and the patient age at the year of the first AF diagnosis was used as entry age. Patients’ attained age at the year of ablation, death, emigration or end of study period was used as exit age. As the exact date of birth was not available for this study, age at the first AF diagnosis was calculated as the difference between the year of the first AF diagnosis and the year of birth. Attained age was calculated as the difference between the year of exit and the year of birth. Only patients aged 25 to 75 at the year of the first AF diagnosis were included in the study. In addition, 80 years was set as an upper age limit for attained age, with patients older than 80 being censored at the year they became 80.

The AF diagnoses were identified from the International Statistical Classification of Diseases and Related Health Problems (ICD-10) diagnosis code: I48 (primary or secondary diagnosis). The code I48 also includes atrial flutter, as it was not possible to distinguish between atrial fibrillation and flutter by diagnosis code before 2013. However, atrial fibrillation is a much more common condition than atrial flutter. The AF ablation procedures were identified from the Nomesco Classification of Surgical Procedures (NCSP) code: (FPB32, FPB22, FPB35, FPB25, FPO25A, FPO10A, FPB13). Patients without an AF diagnosis prior to or at the same date as the AF ablation procedure were excluded.

Educational level was coded applying the international standard classification of education (ISCED) [24]. Larger numbers represented higher educational levels; 0 represented less than primary education, and 8 indicated a doctorate or equivalent while 9 was not classified and regarded as missing. Educational level was recoded into three categories; low (0-2), medium (3-5), and high (6-8), where 3-5 is high school level.

After-tax income was calculated as total income minus assessed tax and negative transfers, with total income representing the sum of income as employee, income from self-employment, property income, capital income, and transfers received. The after-tax income was index-adjusted, to 2015 by the consumer price index (CPI), to account for inflation. From after-tax income a categorical income variable was defined with three categories; low (less than NOK 240 000), medium (NOK 240 000 - 400 000), and high (more than NOK 400 000).

The patients’ hospital referral area was defined by place of residence and the corresponding geographic catchments areas served by the 21 Norwegian hospital trusts (HT). The patients’ regional referral area was defined by the catchment areas for the four regional health authorities (RHA) (North, Central, West and South-East) in Norway. The catchments areas are given by the health authority as administrative borders.

Follow-up time was defined as the number of years from the first AF diagnosis to ablation or censoring. Age, place of residence, income, and educational level were defined according to the date of the first AF diagnosis. Patients with date of censoring equal to date of diagnosis were excluded.

### Statistical analyses

Data were analysed using SAS 9.4 (SAS Institute, Cary NC).

Survival analysis was carried out, separately for females and males, by Cox regression with attained age as time scale. Two models were analysed. In model 1, place of residence was classified by the 21 hospital referral areas (HT). In model 2, place of residence was classified by the four regional referral areas (RHA). Apart from this the two models were identical. Age at the first AF diagnosis was treated as entry age to the study, regarded as left truncation time. AF ablation was considered as the relevant event, with educational level, income level, place of residence (hospital (HT) or regional referral area (RHA)) and follow-up time since the first AF diagnosis as covariates. Follow-up time was time-dependent, while the other covariates were defined by the year of the first AF diagnosis. The categories representing low levels of education and income, Vestre Viken (HT) hospital referral area, South-East (RHA) regional referral area, and follow-up time within the first year were set as reference categories. Vestre Viken HT and South-East RHA have the largest number of AF patients in hospital (HT) and regional referral areas (RHA), respectively. The Efron method was applied for handling ties.

In the initial analysis, travel time to hospital was included as a covariate. Two different measures of travel time were applied, travel time to nearest hospital and travel time to nearest ablation hospital. Travel time was measured as travel time by road from municipality centre. Including travel time as a covariate did not have any impact on the remaining results, and this variable was therefore not included in the analysis.

The proportional hazard assumption was tested by generating time dependent covariates by including interactions of the predictors (education, income and place of residence) and the log of attained age in the model, as described in Allison [25], where significant interaction terms indicate non-proportional hazards.

Spearman’s rank correlation coefficients were computed in order to investigate associations between the variables representing age group, education and income, separately by gender.

In addition, separate analyses for three different time periods were conducted, for the period before the presumed capacity increase (2008-2010), for the period after the instructed capacity increase (2011-2017), and the period 2013-2017. From 2013, it was possible to distinguish between atrial fibrillation and atrial flutter by ICD-10 codes. Only atrial fibrillation patients with diagnosis codes I48.0, I48.1, and I48.2 were included in the analysis for the period 2013-2017.

## Results

### Patient selection and characteristics

**Fig. 1:**
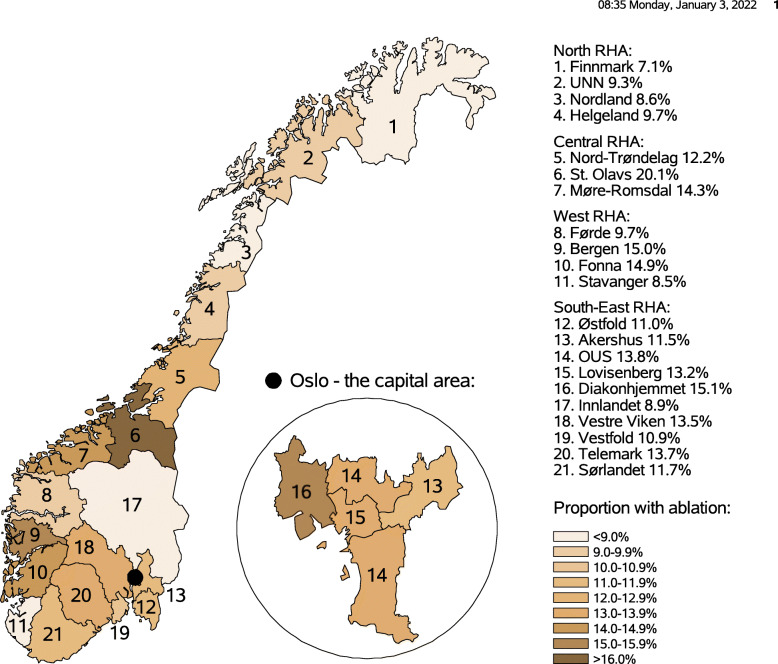
Proportion of atrial fibrillation patients treated with ablation in the 21 hospital referral areas (HTs) in Norway

During 2008-2017, a total of 88 534 patients aged 25–75 years were diagnosed with AF, 29 233 women (mean age at diagnosis 64.6 years) and 59 301 men (mean age at diagnosis 63.0 years) (Table 1). A total of 10 725 patients were treated with ablation in the period, 2 759 women (mean age at ablation 61.1 years) and 7 966 men (mean age at ablation 59.5 years). While 67.0 % of the AF patients were males, 74.3 % of the ablation patients were males. More than half of the AF patients (51.1%) were in the age group 70-80, compared to only 17.6% of the ablation patients in the age group 70-80. Among the AF patients, 27.1% were in the high educational level group, compared to 37.3% among the ablation patients. Of the AF patients, 22.7% belonged to the high income group, compared to 38.8% of the ablation patients. Figure 1 and Table 1 shows the proportion of AF patients treated with ablation in the hospital referral areas. AF patients in Finnmark HT hospital referral area had the lowest proportion (7.1%) treated with ablation, while AF patients in St. Olavs HT hospital referral area had the highest proportion (20.1%).

**Table 1:** Characteristics of AF patients and ablation patients. Norway, 2008-2017

	Atrial fibrillation			Ablation (% proportion with ablation)		
	Female	Male	Total	Female	Male	Total
**Number of patients**	29 233	59 301	88 534	2 759 (9.4%)	7 966 (13.4%)	10 725 (12.1%)
Age at diagnosis, mean [SD]	64.6 [9.9]	63.0 [9.8]	63.6 [9.8]	59.1 [10.6]	57.7 [10.0]	58.0 [10.2]
Age at exit *†*, mean [SD]	68.1 [10.4]	66.7 [10.3]	67.1 [10.4]	61.1 [11.1]	59.5 [10.2]	59.9 [10.5]
Years to exit *†*, mean [SD]	3.5 [2.7]	3.6 [2.8]	3.6 [2.8]	1.9 [2.1]	1.9 [2.1]	1.9 [2.1)
**Age group** *‡*						
25-49	2 041	4 458	6 499	430 (21.1%)	1 283 (28.8%)	1 713 (26.4%)
50-59	2 879	7 867	10 746	550 (19.1%)	2 204 (28.0%)	2 754 (25.6%)
60-69	7 651	18 359	26 010	1 143 (14.9%)	3 224 (17.6%)	4 367 (16.8%)
70-80	16 662	28 617	45 279	636 (3.8%)	1 255 (4.4%)	1 891 (4.2%)
**Education** *‡*						
Low	8 580	13 232	21 812	563 (6.6%)	1 142 (8.6%)	1 705 (7.8%)
Medium	13 643	29 122	42 765	1 313 (9.6%)	3 705 (12.7%)	5 018 (11.7%)
High	7 010	16 947	23 957	883 (12.6%)	3 119 (18.4%)	4 002 (16.7%)
**Income** *‡*						
Low	14 315	10 861	25 176	1 004 (7.0%)	745 (6.9%)	1 749 (6.9%)
Medium	12 156	31 108	43 264	1 339 (11.0%)	3 506 (11.3%)	4 845 (11.2%)
High	2 762	17 332	20 094	416 (15.1%)	3 715 (21.4%)	4 131 (20.6%)
**Hospital referral area (HT)** *‡*∗						
Finnmark (N)	326	847	1 173	15 (4.6%)	68 (8.0%)	83 (7.1%)
UNN (N) ±	977	2 183	3 160	55 (5.6%)	239 (10.9%)	294 (9.3%)
Nordland (N)	765	1 714	2 479	36 (4.7%)	178 (10.4%)	214 (8.6%)
Helgeland (N)	528	1 086	1 614	45 (8.5%)	112 (10.3%)	157 (9.7%)
Nord-Trøndelag (C)	732	1 555	2 287	66 (9.0%)	214 (13.8%)	280 (12.2%)
St. Olavs (C) ±	1 390	3 188	4 578	211 (15.2%)	707 (22.2%)	918 (20.1%)
Møre-Romsdal (C)	1 254	2 809	4 063	158 (12.6%)	425 (15.1%)	583 (14.3%)
Førde (W)	590	1 350	1 940	48 (8.1%)	140 (10.4%)	188 (9.7%)
Bergen (W) ±	1 959	4 472	6 431	223 (11.4%)	744 (16.6%)	967 (15.0%)
Fonna (W)	924	2 098	3 022	115 (12.4%)	335 (16.0%)	450 (14.9%)
Stavanger (W)	3 326	5 180	8 506	198 (6.0%)	526 (10.2%)	724 (8.5%)
Østfold (SE)	1 689	3 391	5 080	144 (8.5%)	417 (12.3%)	561 (11.0%)
Akershus (SE) ∓	2 808	5 253	8 061	257 (9.2%)	673 (12.8%)	930 (11.5%)
OUS (SE) ±	1 224	2 597	3 821	131 (10.7%)	398 (15.3%)	529 (13.8%)
Lovisenberg (SE)	368	775	1 143	45 (12.2%)	106 (13.7%)	151 (13.2%)
Diakonhjemmet (SE)	613	1 378	1 991	70 (11.4%)	231 (16.8%)	301 (15.1%)
Innlandet (SE)	2 497	4 992	7 489	179 (7.2%)	487 (9.8%)	666 (8.9%)
Vestre Viken (SE)	2 884	5 714	8 598	334 (11.6%)	825 (14.4%)	1 159 (13.5%)
Vestfold (SE)	1 567	2 975	4 542	139 (8.9%)	358 (12.0%)	497 (10.9%)
Telemark (SE)	1 103	2 302	3 405	125 (11.3%)	343 (14.9%)	468 (13.7%)
Sørlandet (SE)	1 709	3 442	5 151	165 (9.7%)	440 (12.8%)	605 (11.7%)

^*†*^Exit is ablation, death, emigration or end of study period. For ablation patients the exit is ablation. *‡* At the time of exit. ∗ The four regional health authorities: *N* North, *C* Central, *W* West and *SE* South-East. ± Hospital trust (HT) with ablation centre. ∓ Location of private ablation centre

### Results from statistical analysis

**Fig. 2:**
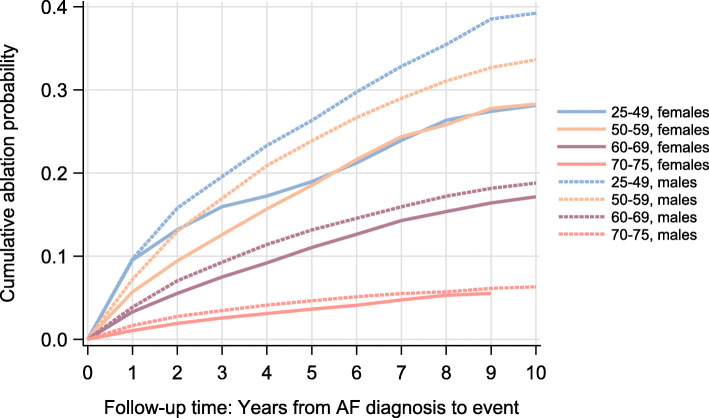
Cumulative ablation probability by follow-up time, separate by age groups (age at AF diagnosis) and gender. Smooth probability curves

Figure 2 shows that a higher proportion of male AF patients were treated with ablation compared to female AF patients, and this was consistent in all age groups and follow-up years. However, the gender differences decreased with increasing age, and in the age groups 60-69 and 70-75 the differences were small.

The rate of ablation, in both female and male AF patients, increased with increasing levels of education. The effect of education was stronger in males than females. Patients with high level of education had around 60% (males) and 35% (females) higher rate of ablation, compared to patients with low education (Table 2).

**Table 2:** Multivariable Cox regression, separate by gender. Hazard ratios (95% confidence interval), adjusted for follow-up time

	Model 1 (HT)			Model 2 (RHA)	
	Female	Male		Female	Male
**Education**			**Education**		
Low	1.0 (ref)	1.0 (ref)	Low	1.0 (ref)	1.0 (ref)
Medium	1.31 (1.19 - 1.45)	1.28 (1.20 - 1.37)	Medium	1.32 (1.19 - 1.45)	1.29 (1.21 - 1.38)
High	1.34 (1.19 - 1.51)	1.62 (1.51 - 1.74)	High	1.36 (1.21 - 1.53)	1.67 (1.55 - 1.79)
**Income**			**Income**		
Low	1.0 (ref)	1.0 (ref)	Low	1.0 (ref)	1.0 (ref)
Medium	1.20 (1.10 - 1.31)	1.54 (1.42 - 1.67)	Medium	1.20 (1.10 - 1.31)	1.54 (1.42 - 1.67)
High	1.40 (1.23 - 1.59)	1.84 (1.69 - 2.00)	High	1.37 (1.21 - 1.56)	1.82 (1.68 - 1.97)
**Follow-up time (years)**			**Follow-up time (years)**		
1	1.0 (ref)	1.0 (ref)	1	1.0 (ref)	1.0 (ref)
2	0.68 (0.61 - 0.76)	0.86 (0.81 - 0.91)	2	0.68 (0.61 - 0.75)	0.85 (0.80 - 0.91)
3	0.63 (0.56 - 0.71)	0.64 (0.60 - 0.69)	3	0.63 (0.56 - 0.71)	0.64 (0.59 - 0.69)
4	0.58 (0.51 - 0.67)	0.70 (0.65 - 0.75)	4	0.58 (0.50 - 0.66)	0.70 (0.64 - 0.75)
5	0.68 (0.59 - 0.79)	0.62 (0.56 - 0.68)	5	0.68 (0.58 - 0.79)	0.62 (0.56 - 0.67)
6	0.73 (0.61 - 0.86)	0.63 (0.57 - 0.70)	6	0.73 (0.61 - 0.87)	0.63 (0.57 - 0.70)
7	0.85 (0.71 - 1.03)	0.63 (0.56 - 0.71)	7	0.86 (0.71 - 1.03)	0.63 (0.56 - 0.71)
8	0.63 (0.49 - 0.82)	0.62 (0.53 - 0.71)	8	0.64 (0.49 - 0.82)	0.62 (0.53 - 0.72)
9	0.62 (0.45 - 0.86)	0.59 (0.49 - 0.71)	9	0.63 (0.45 - 0.86)	0.59 (0.49 - 0.71)
10 or more	0.37 (0.21 - 0.66)	0.33 (0.24 - 0.46)	10 or more	0.37 (0.21 - 0.66)	0.33 (0.24 - 0.46)
**Hospital referral area (HT)**			**Regional referral area (RHA)**		
Finnmark (N)	0.39 (0.23 - 0.66)	0.62 (0.48 - 0.80)	North	0.61 (0.52 - 0.72)	0.83 (0.76 - 0.90)
UNN (N)	0.52 (0.39 - 0.69)	0.81 (0.70 - 0.94)	Central	1.43 (1.29 - 1.59)	1.52 (1.43 - 1.62)
Nordland (N)	0.45 (0.32 - 0.63)	0.82 (0.70 - 0.97)	West	0.79 (0.72 - 0.87)	1.01 (0.95 - 1.07)
Helgeland (N)	0.73 (0.53 - 1.00)	0.80 (0.66 - 0.97)	South-East	1.0 (ref)	1.0 (ref)
Nord-Trøndelag (C)	0.86 (0.66 - 1.12)	1.14 (0.98 - 1.32)			
St. Olavs (C)	1.50 (1.26 - 1.78)	1.78 (1.61 - 1.97)			
Møre-Romsdal (C)	1.19 (0.99 - 1.44)	1.24 (1.10 - 1.39)			
Førde (W)	0.81 (0.60 - 1.10)	0.83 (0.70 - 1.00)			
Bergen (W)	1.09 (0.92 - 1.29)	1.27 (1.15 - 1.40)			
Fonna (W)	1.18 (0.96 - 1.47)	1.29 (1.13 - 1.46)			
Stavanger (W)	0.38 (0.32 - 0.46)	0.65 (0.58 - 0.72)			
Østfold (SE)	0.84 (0.69 - 1.02)	0.95 (0.85 - 1.07)			
Akershus (SE)	0.81 (0.69 - 0.96)	0.94 (0.85 - 1.04)			
OUS (SE)	0.90 (0.73 - 1.10)	1.00 (0.89 - 1.13)			
Lovisenberg (SE)	0.97 (0.71 - 1.32)	0.90 (0.73 - 1.10)			
Diakonhjemmet (SE)	0.91 (0.70 - 1.17)	1.02 (0.88 - 1.18)			
Innlandet (SE)	0.67 (0.56 - 0.81)	0.80 (0.71 - 0.89)			
Vestre Viken (SE)	1.0 (ref)	1.0 (ref)			
Vestfold (SE)	0.78 (0.64 - 0.95)	0.87 (0.77 - 0.99)			
Telemark (SE)	1.05 (0.86 - 1.29)	1.13 (1.00 - 1.28)			
Sørlandet (SE)	0.90 (0.74 - 1.08)	0.98 (0.87 - 1.10)			
**Interactions with attained age (p-values)**			**Interactions with attained age (p-values)**		
Education	<0.001	<0.001	Education	<0.001	0.001
Income	0.53	0.17	Income	0.85	0.06
Area (HT)	<0.001	0.50	Area (RHA)	<0.001	0.25

The rate of ablation in AF patients also increased with increasing levels of income. Similarly, as for education, the effect of income was stronger in males than females, with around 80% (males) and 40% (females) higher rate of ablation in patients with high income, compared to patients of the same gender with low income.

Compared to patients living in the regional referral area of the South-East RHA, patients living in the regional referral area of the Central RHA had around 50% higher rates of ablation, and patients living in the regional referral area of the North RHA had lower rates of ablation (39% lower for females and 17% lower for males). There was substantial variation within the RHAs. Patients living in the four hospital referral areas in the North RHA all had lower rates of ablation, compared to patients living in the hospital referral area of Vestre Viken HT. Patients living in the hospital referral area of St. Olavs HT, in the Central RHA, had the highest rates of ablation in the country, and around three times as high ablation rates, compared to patients living in the hospital referral area of Finnmark HT (3.9 times higher in females and 2.9 times higher in males).

The rate of ablation decreased with increasing number of years since AF diagnosis in both males and females in both models; however, the decreasing trend was not consistent throughout all the follow-up years.

The tests for proportional hazard showed significant interactions with attained age for education in both females and males and place of residence in females (Table 2). Thus, the effects of education in both genders and the effects of place of residence in females may differ over age groups. This was confirmed by the results from multivariable Cox regressions, separate by both gender and age groups (Tables 3 and 4). In females, the positive effect of education was found in the age groups 50-59, 60-69 and 70-75, and the effect of high educational level increased with increasing age. In contrast, there was no effect in the age group 25-49. In males, the positive education effect was found in all age groups, and the effect of high educational level increased with increasing age.

**Table 3:** Multivariable Cox regression, Model 2 (RHA), adjusted for follow-up time. Separate by gender and age groups (age at AF diagnosis). Hazard ratios (95% confidence interval)

	All	25-49	50-59	60-69	70-75
**Female**					
***Education***					
Low	1.0 (ref)	1.0 (ref)	1.0 (ref)	1.0 (ref)	1.0 (ref)
Medium	1.32 (1.19 - 1.45)	1.21 (0.94 - 1.57)	1.28 (1.04 - 1.57)	1.28 (1.11 - 1.49)	1.54 (1.19 - 1.98)
High	1.36 (1.21 - 1.53)	1.01 (0.77 - 1.33)	1.28 (1.02 - 1.61)	1.54 (1.29 - 1.84)	1.63 (1.17 - 2.27)
***Income***					
Low	1.0 (ref)	1.0 (ref)	1.0 (ref)	1.0 (ref)	1.0 (ref)
Medium	1.20 (1.10 - 1.31)	1.14 (0.90 - 1.44)	1.31 (1.09 - 1.58)	1.13 (0.99 - 1.28)	1.23 (0.98 - 1.55)
High	1.37 (1.21 - 1.56)	1.45 (1.09 - 1.93)	1.35 (1.05 - 1.73)	1.33 (1.09 - 1.62)	1.60 (1.01 - 2.55)
***Regional referral area (RHA)***					
North	0.61 (0.52 - 0.72)	0.51 (0.34 - 0.77)	0.60 (0.43 - 0.84)	0.65 (0.51 - 0.84)	0.69 (0.44 - 1.08)
Central	1.43 (1.29 - 1.59)	1.01 (0.75 - 1.38)	1.15 (0.92 - 1.44)	1.67 (1.44 - 1.95)	1.86 (1.41 - 2.45)
West	0.79 (0.72 - 0.87)	0.33 (0.27 - 0.42)	0.74 (0.61 - 0.90)	1.10 (0.95 - 1.27)	1.35 (1.05 - 1.74)
South-East	1.0 (ref)	1.0 (ref)	1.0 (ref)	1.0 (ref)	1.0 (ref)
**Male**					
***Education***					
Low	1.0 (ref)	1.0 (ref)	1.0 (ref)	1.0 (ref)	1.0 (ref)
Medium	1.29 (1.21 - 1.38)	1.37 (1.18 - 1.60)	1.25 (1.12 - 1.41)	1.32 (1.18 - 1.47)	1.23 (0.99 - 1.52)
High	1.67 (1.55 - 1.79)	1.45 (1.23 - 1.70)	1.52 (1.34 - 1.72)	1.82 (1.62 - 2.05)	2.17 (1.72 - 2.73)
***Income***					
Low	1.0 (ref)	1.0 (ref)	1.0 (ref)	1.0 (ref)	1.0 (ref)
Medium	1.54 (1.42 - 1.67)	1.55 (1.28 - 1.88)	1.72 (1.48 - 2.01)	1.39 (1.23 - 1.56)	1.50 (1.20 - 1.88)
High	1.82 (1.68 - 1.97)	1.64 (1.34 - 1.99)	2.05 (1.76 - 2.39)	1.68 (1.49 - 1.91)	1.92 (1.47 - 2.50)
***Regional referral area (RHA)***					
North	0.83 (0.76 - 0.90)	0.87 (0.72 - 1.06)	0.83 (0.72 - 0.96)	0.80 (0.69 - 0.92)	0.79 (0.58 - 1.07)
Central	1.52 (1.43 - 1.62)	1.50 (1.30 - 1.73)	1.47 (1.31 - 1.64)	1.52 (1.38 - 1.67)	1.78 (1.47 - 2.17)
West	1.01 (0.95 - 1.07)	0.81 (0.71 - 0.92)	0.87 (0.78 - 0.96)	1.20 (1.10 - 1.31)	1.29 (1.07 - 1.55)
South-East	1.0 (ref)	1.0 (ref)	1.0 (ref)	1.0 (ref)	1.0 (ref)

**Table 4:** Multivariable Cox regression, Model 1 (HT), adjusted for follow-up time. Separate by gender and age groups (age at AF diagnosis). Hazard ratios (95% confidence interval)

	All	25-49	50-59	60-69	70-75
**Female**					
***Education***					
Low	1.0 (ref)	1.0 (ref)	1.0 (ref)	1.0 (ref)	1.0 (ref)
Medium	1.31 (1.19 - 1.45)	1.23 (0.95 - 1.60)	1.27 (1.03 - 1.56)	1.28 (1.10 - 1.48)	1.51 (1.17 - 1.94)
High	1.34 (1.19 - 1.51)	1.01 (0.77 - 1.33)	1.26 (1.00 - 1.59)	1.53 (1.28 - 1.83)	1.56 (1.12 - 2.18)
**Income**					
Low	1.0 (ref)	1.0 (ref)	1.0 (ref)	1.0 (ref)	1.0 (ref)
Medium	1.20 (1.10 - 1.31)	1.15 (0.91 - 1.46)	1.33 (1.11 - 1.60)	1.13 (0.99 - 1.29)	1.20 (0.95 - 1.51)
High	1.40 (1.23 - 1.59)	1.52 (1.14 - 2.03)	1.42 (1.10 - 1.82)	1.34 (1.10 - 1.64)	1.54 (0.96 - 2.46)
***Hospital referral area (HT)***					
Finnmark (N)	0.39 (0.23 - 0.66)	0.44 (0.14 - 1.40)	0.54 (0.22 - 1.32)	0.29 (0.12 - 0.71)	0.43 (0.10 - 1.80)
UNN (N)	0.52 (0.39 - 0.69)	0.72 (0.39 - 1.34)	0.62 (0.35 - 1.10)	0.42 (0.27 - 0.66)	0.52 (0.23 - 1.16)
Nordland (N)	0.45 (0.32 - 0.63)	0.33 (0.10 - 1.05)	0.52 (0.26 - 1.04)	0.44 (0.27 - 0.72)	0.53 (0.23 - 1.25)
Helgeland (N)	0.73 (0.53 - 1.00)	0.29 (0.13 - 0.67)	0.68 (0.34 - 1.35)	1.08 (0.70 - 1.66)	0.90 (0.38 - 2.12)
Nord-Trøndelag (C)	0.86 (0.66 - 1.12)	0.67 (0.32 - 1.40)	0.99 (0.58 - 1.70)	0.90 (0.62 - 1.29)	0.65 (0.28 - 1.54)
St. Olavs (C)	1.50 (1.26 - 1.78)	0.92 (0.56 - 1.51)	1.32 (0.91 - 1.91)	1.61 (1.25 - 2.06)	2.23 (1.45 - 3.43)
Møre-Romsdal (C)	1.19 (0.99 - 1.44)	1.06 (0.63 - 1.77)	1.02 (0.69 - 1.52)	1.32 (1.01 - 1.73)	1.34 (0.79 - 2.26)
Førde (W)	0.81 (0.60 - 1.10)	0.41 (0.15 - 1.12)	0.82 (0.43 - 1.54)	0.94 (0.62 - 1.43)	0.84 (0.38 - 1.87)
Bergen (W)	1.09 (0.92 - 1.29)	0.82 (0.52 - 1.27)	1.18 (0.83 - 1.67)	1.00 (0.78 - 1.29)	1.57 (1.02 - 2.41)
Fonna (W)	1.18 (0.96 - 1.47)	0.78 (0.44 - 1.36)	1.18 (0.74 - 1.88)	1.30 (0.96 - 1.76)	1.38 (0.79 - 2.44)
Stavanger (W)	0.38 (0.32 - 0.46)	0.20 (0.14 - 0.29)	0.43 (0.30 - 0.63)	0.59 (0.44 - 0.78)	0.71 (0.41 - 1.22)
Østfold (SE)	0.84 (0.69 - 1.02)	0.71 (0.43 - 1.17)	0.92 (0.62 - 1.38)	0.90 (0.68 - 1.19)	0.65 (0.36 - 1.18)
Akershus (SE)	0.81 (0.69 - 0.96)	0.91 (0.62 - 1.33)	0.85 (0.61 - 1.19)	0.76 (0.60 - 0.97)	0.80 (0.50 - 1.27)
OUS (SE)	0.90 (0.73 - 1.10)	0.82 (0.52 - 1.31)	1.21 (0.82 - 1.79)	0.77 (0.56 - 1.06)	0.89 (0.50 - 1.59)
Lovisenberg (SE)	0.97 (0.71 - 1.32)	1.16 (0.65 - 2.08)	0.54 (0.23 - 1.23)	1.09 (0.70 - 1.69)	0.56 (0.13 - 2.30)
Diakonhjemmet (SE)	0.91 (0.70 - 1.17)	1.09 (0.60 - 1.99)	0.82 (0.46 - 1.45)	0.70 (0.46 - 1.06)	1.58 (0.88 - 2.82)
Innlandet (SE)	0.67 (0.56 - 0.81)	0.79 (0.52 - 1.21)	0.98 (0.69 - 1.37)	0.48 (0.36 - 0.65)	0.74 (0.44 - 1.22)
Vestre Viken (SE)	1.0 (ref)	1.0 (ref)	1.0 (ref)	1.0 (ref)	1.0 (ref)
Vestfold (SE)	0.78 (0.64 - 0.95)	0.55 (0.33 - 0.93)	0.96 (0.66 - 1.40)	0.76 (0.56 - 1.02)	0.88 (0.51 - 1.52)
Telemark (SE)	1.05 (0.86 - 1.29)	1.23 (0.78 - 1.93)	1.12 (0.74 - 1.69)	0.99 (0.73 - 1.36)	0.81 (0.44 - 1.52)
Sørlandet (SE)	0.90 (0.74 - 1.08)	0.92 (0.61 - 1.41)	1.23 (0.86 - 1.77)	0.78 (0.58 - 1.04)	0.74 (0.43 - 1.29)
**Male**					
***Education***					
Low	1.0 (ref)	1.0 (ref)	1.0 (ref)	1.0 (ref)	1.0 (ref)
Medium	1.28 (1.20 - 1.37)	1.34 (1.15 - 1.56)	1.25 (1.11 - 1.40)	1.30 (1.17 - 1.45)	1.22 (0.99 - 1.52)
High	1.62 (1.51 - 1.74)	1.40 (1.19 - 1.65)	1.48 (1.30 - 1.68)	1.78 (1.58 - 2.00)	2.12 (1.68 - 2.68)
***Income***					
Low	1.0 (ref)	1.0 (ref)	1.0 (ref)	1.0 (ref)	1.0 (ref)
Medium	1.54 (1.42 - 1.67)	1.58 (1.30 - 1.92)	1.72 (1.48 - 2.01)	1.38 (1.23 - 1.56)	1.48 (1.18 - 1.85)
High	1.84 (1.69 - 2.00)	1.69 (1.39 - 2.06)	2.07 (1.77 - 2.41)	1.69 (1.49 - 1.91)	1.86 (1.42 - 2.43)
***Hospital referral area (HT)***					
Finnmark (N)	0.62 (0.48 - 0.80)	0.86 (0.49 - 1.52)	0.49 (0.31 - 0.78)	0.60 (0.41 - 0.88)	0.88 (0.42 - 1.81)
UNN (N)	0.81 (0.70 - 0.94)	1.12 (0.81 - 1.53)	0.92 (0.73 - 1.17)	0.66 (0.51 - 0.84)	0.55 (0.32 - 0.94)
Nordland (N)	0.82 (0.70 - 0.97)	1.01 (0.69 - 1.47)	0.89 (0.68 - 1.18)	0.73 (0.57 - 0.95)	0.62 (0.34 - 1.11)
Helgeland (N)	0.80 (0.66 - 0.97)	0.61 (0.36 - 1.04)	0.88 (0.63 - 1.22)	0.81 (0.59 - 1.10)	0.80 (0.41 - 1.54)
Nord-Trøndelag (C)	1.14 (0.98 - 1.32)	1.45 (1.04 - 2.01)	1.23 (0.94 - 1.62)	0.94 (0.74 - 1.20)	1.09 (0.67 - 1.79)
St. Olavs (C)	1.78 (1.61 - 1.97)	1.87 (1.47 - 2.39)	1.75 (1.46 - 2.09)	1.70 (1.45 - 1.99)	1.99 (1.46 - 2.70)
Møre-Romsdal (C)	1.24 (1.10 - 1.39)	1.46 (1.10 - 1.93)	1.30 (1.06 - 1.60)	1.13 (0.94 - 1.35)	1.10 (0.75 - 1.62)
Førde (W)	0.83 (0.70 - 1.00)	1.16 (0.77 - 1.74)	0.72 (0.52 - 1.01)	0.81 (0.61 - 1.07)	0.80 (0.46 - 1.38)
Bergen (W)	1.27 (1.15 - 1.40)	1.25 (0.98 - 1.59)	1.21 (1.01 - 1.44)	1.30 (1.12 - 1.52)	1.32 (0.97 - 1.80)
Fonna (W)	1.29 (1.13 - 1.46)	1.38 (1.01 - 1.89)	1.15 (0.91 - 1.46)	1.31 (1.08 - 1.59)	1.39 (0.93 - 2.07)
Stavanger (W)	0.65 (0.58 - 0.72)	0.61 (0.48 - 0.77)	0.60 (0.49 - 0.73)	0.75 (0.63 - 0.90)	0.74 (0.50 - 1.09)
Østfold (SE)	0.95 (0.85 - 1.07)	1.09 (0.83 - 1.44)	0.98 (0.80 - 1.21)	0.83 (0.69 - 1.01)	1.07 (0.74 - 1.54)
Akershus (SE)	0.94 (0.85 - 1.04)	1.13 (0.90 - 1.44)	1.00 (0.84 - 1.20)	0.82 (0.69 - 0.97)	0.87 (0.63 - 1.21)
OUS (SE)	1.00 (0.89 - 1.13)	1.39 (1.08 - 1.80)	1.03 (0.83 - 1.27)	0.86 (0.71 - 1.05)	0.87 (0.58 - 1.31)
Lovisenberg (SE)	0.90 (0.73 - 1.10)	0.83 (0.55 - 1.24)	1.00 (0.70 - 1.44)	1.05 (0.76 - 1.46)	0.49 (0.18 - 1.33)
Diakonhjemmet (SE)	1.02 (0.88 - 1.18)	1.08 (0.76 - 1.53)	1.07 (0.80 - 1.42)	0.95 (0.76 - 1.18)	1.05 (0.68 - 1.62)
Innlandet (SE)	0.80 (0.71 - 0.89)	0.94 (0.72 - 1.23)	0.85 (0.70 - 1.04)	0.74 (0.62 - 0.88)	0.62 (0.42 - 0.90)
Vestre Viken (SE)	1.0 (ref)	1.0 (ref)	1.0 (ref)	1.0 (ref)	1.0 (ref)
Vestfold (SE)	0.87 (0.77 - 0.99)	0.96 (0.71 - 1.30)	1.05 (0.86 - 1.29)	0.75 (0.62 - 0.92)	0.61 (0.39 - 0.94)
Telemark (SE)	1.13 (1.00 - 1.28)	1.27 (0.95 - 1.69)	1.21 (0.97 - 1.51)	1.11 (0.91 - 1.35)	0.73 (0.45 - 1.16)
Sørlandet (SE)	0.98 (0.87 - 1.10)	1.22 (0.93 - 1.59)	1.08 (0.88 - 1.32)	0.85 (0.70 - 1.02)	0.80 (0.54 - 1.18)

The hazard ratios increased with age in patients living in the West (both females and males) and Central (females) RHA, compared to patients living in the South-East RHA (Table 3). This age effect was also present in females living in Bergen and Fonna HT (both in West RHA), compared to patients living in Vestre Viken HT (Table 4).

The correlation coefficients between covariates were relatively low, except for the correlation between education and income (see supplementary Table A1). Cox regression analysis without SES adjustment gave similar results for the HTs and RHAs as in Table 2 (see supplementary, Table A2). The results from the analysis of the three different periods were generally similar to the results in the main analysis (see supplementary).

## Discussion

### Principal findings

Our data show substantial socioeconomic and geographic differences in frequency of ablation therapy in patients with diagnosed atrial fibrillation in Norway. AF patients living in the regional referral area of the Central RHA had the highest ablation rates, while patients living in the regional referral area of the North RHA were less likely to receive ablation treatment. AF patients with high level of education and high level of income were more frequently treated with ablation.

### Age

We found a marked age effect, with younger patients being more likely to receive ablation than older patients. The European guidelines for treatment of atrial fibrillation from 2010 recommend ablation for younger patients with symptomatic paroxysmal or persistent atrial fibrillation in whom antiarrhythmic drug treatment failed [4]. In an update from 2012, these indications were further strengthened [5]. The prevalence of AF increases progressively with age, and age is an independent risk factor for adverse outcomes in AF. AF catheter ablation may be an effective and safe option in selected older individuals with success rates comparable to younger patients [26]. However, age may be a predictor of complications in AF ablation [27] and a longer follow-up study suggests an age-related increase in risk of AF recurrence, major adverse cardiac events, and death after ablation [28].

### Gender

Our data showed that females are treated with ablation for atrial fibrillation to a lesser extent than males. This is in line with other studies [29–31]. Females are referred for AF catheter ablation later than males, possibly reflecting AF occurrence later in life among females [32]. Female atrial fibrillation patients more commonly present comorbidities and are referred to hospital care later and with longer disease history [29]. This might affect the clinicians’ decisions concerning therapeutic strategy [29]. A review recommends a gendered management strategy in treating AF, as the gender differences in AF are substantial, and antiarrhythmic drugs and ablation can have more complications in females than in males [30]. However, both the 2020 ESC Guidelines and a recent review recommend that females and males are offered diagnostic assessment and therapies equally [6, 33]. It is difficult to conclude on gender differences in risk and benefit of different treatment strategies in AF patients, as females are significantly underrepresented in studies on AF [30]. However, in Norway, the gender differences seem to diminish with age, as the ablation probabilities are almost equal for the older AF patients.

### Income and education

Both patients with high level of education and patients with high income were more likely to receive ablation than patients with low level of education and low income. These inequalities increased with increasing age. However, no effect of education was found in the youngest females.

Socioeconomic differences in use of health care services have been discussed extensively, also in countries as Norway with universal health care systems where there is no co-payment from the patients for in-hospital treatment. Our finding, that patients with higher education and higher income are over-represented among those who undergo ablation therapy, is in accordance with several other reports of such gradients in the use of specialised health care, both international and from Norway [16, 17, 34]. For coronary heart disease, socioeconomic differences in revascularisation procedures have been reported in several European countries [35–38]. In a study from Denmark, socioeconomic differences were documented in outcomes after hospital admission for atrial fibrillation or flutter, both in mortality and treatment with ablation [39]. A Norwegian study indicated that low SES was related to higher mortality in AF patients [40].

One of the mechanisms underlying SES differences in health care use may be found in the concept of health literacy [41]. Health literacy is the degree to which individuals have the ability to find, understand, and use information and services to inform health-related decisions and actions for themselves and others [42]. Health literate patients may be more capable of understanding, questioning and discussing treatment options with their physician. It has been demonstrated that low functional health literacy is associated with sub-optimal use of health care services [43], and the association between educational level and health literacy is well documented [44]. A systematic review of associations between socioeconomic status, atrial fibrillation, and outcomes found no consistent social gradient in the risk of AF [45]. However, when AF was present there was a social gradient in the risk of poorer outcome. Low SES was associated with outcomes such as poorer treatment, less knowledge, poorer psychological health and higher mortality.

Demand for a specific treatment depends on the preferences, perceptions and prejudices of both patient and health care provider [46]. Two equally healthy individuals may assess their health differently because their conceptions of good health and their health expectations are contingent on their knowledge of disease and available treatments. More highly educated people are reported to assess their health more negatively, and superior information acquisition skills increase the likelihood that they will recognise and report symptoms of disease earlier [19]. The socioeconomic gradient in physical activity is well known, and individuals in higher SES classes are more likely to be physically active compared to individuals in lower SES classes [47, 48]. Even though physical activity improves the health of AF patients, it is also reported that exercise can trigger AF episodes in paroxysmal AF patients [49]. Thus, AF patients in higher SES classes might be more affected by AF, and may therefore both prefer and demand ablation treatment to a greater extent than AF patients in lower SES classes. However, several studies have shown that exercise can reduce the burden of AF [50–52].

### Follow-up time

The rate of ablation decreased with time since the AF diagnosis. This is as expected, as the natural history of AF is characterised by progressive atrial remodelling. Shorter duration between the time of first AF diagnosis and AF ablation is associated with an increased likelihood of ablation procedural success [53]. The atrial substrate and remodelling increase with the duration of ongoing AF and lead to greater resistance to successful AF ablation, and higher AF recurrence rates [53].

### Geographic variation

Substantial geographic variation was found in the probability of ablation according to the patients’ place of residence, both considering hospital referral areas (HT) and regional referral areas (RHA).

Geographic variation in ablation utilisation has been documented in studies from both Europe and the US [54, 55]. Also among Medicare beneficiaries in the US, marked geographic variation in the use of catheter ablation for atrial fibrillation has been found. The variation was not associated with the prevalence of atrial fibrillation, availability of cardiologists or end-of-life resource use [56].

Unwarranted variation in health care is mainly due to services that can be defined as preference-sensitive or supply-sensitive [57]. Preference-sensitive care represents patient preferences, clinical practice, and preferences and beliefs of a single clinician or department rather than a clear evidence-based approach. Supply-sensitive care refers to local capacity of health care resources, such as ablation clinics. The observed geographic variation in this study is probably related to both differences in clinical practice and differences in capacity.

The reasons for the observed variation in the ablation rate are not clear but may reflect provider preferences and uncertainty of safety and/or efficacy of the procedure in a region. Ablation for atrial fibrillation is a procedure that is developing fast. The rapid development in procedural techniques and indications may increase the likelihood that specialists performing the procedure show individual variation in patient selection. Some specialists might primarily select patients without structural heart disease who have highly symptomatic, paroxysmal atrial fibrillation and have failed one or more treatments with antiarrhythmic drugs. Others might have a different threshold and offer ablation as first-line therapy, or to patients with persistent or chronic fibrillation, with or without underlying structural heart disease. Guidelines might be implemented at different time points in the regions, as the shift in ablation probability between age groups in West RHA might be an example of. Furthermore, not all primary care and local hospital physicians, who are responsible for referring the patients to specialists in Norway, may be equally familiar with the potential benefit of the procedure.

Differences in ablation capacity at the five ablation centres can also contribute to the observed geographic variation. The ablation procedure in Norway was first implemented in 2001 in the West RHA, while the North RHA was the last RHA to implement the procedure in 2009. The waiting time for ablation has been more than a year during the study period, despite the fact that all five ablation centres have fully utilised the capacity. However, ablation capacity cannot alone explain the threefold difference in rates of ablation between patients living in the hospital referral areas of Finnmark HT and St. Olavs HT.

Differences in sociodemographic factors between the hospital referral areas might be a source of variation. However, the funding system for public hospitals in Norway is based on a model that accounts for regional differences in sociodemographic factors and differences in the cost of providing specialist health care services. The aim of the model is to ensure equitable health care services across the regions.

### Strengths and limitations

The major strength of this study is that it covers, for all practical purposes, all patients who have been diagnosed with atrial fibrillation within the specialised health care services and all patients who have undergone ablation within the national health care system in Norway during the period 2008-2017. We have information about income and educational level of all patients included in the study. Privately financed ablations are not included, as there are no available data on privately financed procedures in Norway. However, the vast majority of Norwegian health care services are publicly financed, and this is, even more, the case for ablations. Thus, there are no reasons to believe that this limitation is important for the interpretation of our data.

During the study period, the guidelines for treatment of AF patients have evolved, and the results should be interpreted in accordance with the applicable guidelines at any given time. The ICD-10 code I48 for atrial fibrillation was used to identify the patient population in this study. A possible limitation is that this also includes atrial flutter. Until 2013 it was not possible to distinguish between atrial fibrillation and atrial flutter. This means that the actual number of atrial fibrillation patients is somewhat lower than reported. However, the separate analysis for the period 2013-2017, with atrial fibrillation patients only, showed similar associations.

Individuals moving between residential areas within the study period could be a limitation. However, this will probably have a small effect, since the study population is older and less people tend to move compared to younger people.

The test for the proportional hazard assumption and the separate analysis for age groups showed that some of the effects varied over age groups. Interpretation of the results must take this age effect into consideration.

## Conclusion

This study demonstrates a significant socioeconomic gradient in the proportion of AF patients treated with ablation in Norway. This gradient is probably related to both differences in health literacy and differences in patient preference and demands between socioeconomic groups. Further research exploring the mechanisms by which SES influences the choice of treatment of AF patients is warranted. A substantial part of the geographic variation is probably related to differences in capacity. However, geographic variation caused by differences in clinical practice and provider preferences implies a need for clearer guidelines, both at specialist level and also at the referring level. The observed gender differences in ablation probabilities, especially in younger AF patients, do not necessarily reflect differences in AF morbidity only but also differences in clinical strategies. More research on gender differences in the effect of treatment strategies is needed.

## Supplementary Information

**Additional file 1** Supplementary tables.

## Data Availability

The data that support the findings of this study are available from the Norwegian Patient Registry (NPR) and the Statistics Norway (SSB), but restrictions apply to the availability of these data, which were used under license for the current study, and so are not publicly available. Data are however available from the authors upon reasonable request and with permission of the NPR and the SSB.

## Abbreviations

AF: Atrial fibrillation
HT: Hospital trust
ICD-10: International Statistical Classification of Diseases and Related Health Problems diagnosis codes
ISCED: International standard classification of education
NCSP: Nomesco Classification of Surgical Procedures codes NPR: Norwegian Patient Registry
RHA: Regional health authority
SES: Socioeconomic status
SSB: Statistics Norway
