# Correction to: Socioeconomic and geographic differences in ablation of atrial fibrillation in Norway - a national cohort study

**DOI:** 10.1186/s12889-022-13011-4

**Published:** 2022-04-11

**Authors:** Frank Olsen, Bård Uleberg, Bjarne K. Jacobsen, Ivar Heuch, Pål M. Tande, Einar Bugge, Lise Balteskard

**Affiliations:** 1 Department of Community Medicine, UiT The Arctic University of Norway, Tromsø, Norway; 2 Centre for Clinical Documentation and Evaluation (SKDE), Northern Norway Regional Health Authority, Tromsø, Norway; 3 Centre for Sami Health Research, UiT The Arctic University of Norway, Tromsø, Norway; 4 Department of Mathematics, University of Bergen, Bergen, Norway; 5 Department of Cardiology, University Hospital of North Norway, Tromsø, Norway; 6 Department of Clinical Medicine, UiT The Arctic University of Norway, Tromsø, Norway; 7 Centre for Clinical Research and Education, University Hospital of North Norway, Tromsø, Norway

**Correction to: BMC Public Health 22, 303 (2022)**

**https://doi.org/10.1186/s12889-022-12628-9**

Following publication of the original article, the authors identified an error in a table 1 footnote and a typo in the age group of table 1 and the results section. The incorrect and correct information is listed below. The other elements of the table remain the same, and the interpretation of the results remains unchanged. The original article has been updated.

**Incorrect footnote**

‡ At the time of diagnosis

**Correct footnote**

‡ At the time of exit

**Incorrect**

More than half of the AF patients (51.1%) were in the age group 70-75, compared to only 17.6% of the ablation patients in the age group 70-75.

**Correct**

More than half of the AF patients (51.1%) were in the age group 70-80, compared to only 17.6% of the ablation patients in the age group 70-80.

