# Supplementary material for: Socioeconomic and geographic differences in ablation of atrial fibrillation in Norway - a national cohort study
Source: BMC Public Health. 2022 Feb 14;22:303. doi: 10.1186/s12889-022-12628-9 (PMC8842863; doi:10.1186/s12889-022-12628-9)
Supplement: Supplementary file 1 — Additional file 1 Supplementary tables. [file 12889_2022_12628_MOESM1_ESM.pdf]

Supplementary

Table A1: Correlations, Spearman rank correlation.

		Age group	Education	Income
Females	Age group	1	-0.164	-0.265
	Education	-0.164	1	0.430
	Income	-0.265	0.430	1
Males	Age group	1	-0.071	-0.232
	Education	-0.071	1	0.376
	Income	-0.232	0.376	1

Table A2: Multivariable Cox regression without SES, separate by gender. Hazard ratios (95% confidence interval), adjusted for follow-up time.

	Model 1 (HT)			Model 2 (RHA)	
	Female	Male		Female	Male
**Follow-up time (years)**			**Follow-up time (years)**		
1	1.0 (ref)	1.0 (ref)	1	1.0 (ref)	1.0 (ref)
2	0.68 (0.61 - 0.76)	0.86 (0.81 - 0.91)	2	0.68 (0.61 - 0.76)	0.85 (0.80 - 0.91)
3	0.63 (0.56 - 0.72)	0.64 (0.60 - 0.69)	3	0.63 (0.56 - 0.71)	0.64 (0.59 - 0.69)
4	0.59 (0.51 - 0.67)	0.70 (0.65 - 0.75)	4	0.58 (0.51 - 0.67)	0.70 (0.64 - 0.75)
5	0.68 (0.59 - 0.79)	0.61 (0.56 - 0.67)	5	0.68 (0.59 - 0.79)	0.61 (0.56 - 0.67)
6	0.73 (0.61 - 0.86)	0.63 (0.56 - 0.70)	6	0.73 (0.61 - 0.87)	0.63 (0.56 - 0.70)
7	0.85 (0.71 - 1.03)	0.62 (0.55 - 0.71)	7	0.85 (0.71 - 1.03)	0.63 (0.55 - 0.71)
8	0.63 (0.49 - 0.82)	0.61 (0.53 - 0.71)	8	0.64 (0.49 - 0.82)	0.62 (0.53 - 0.72)
9	0.63 (0.45 - 0.86)	0.59 (0.49 - 0.71)	9	0.63 (0.46 - 0.87)	0.59 (0.49 - 0.71)
10 or more	0.38 (0.21 - 0.67)	0.33 (0.24 - 0.46)	10 or more	0.38 (0.21 - 0.67)	0.34 (0.24 - 0.47)
**Hospital referral area (HT)**			**Regional referral area (RHA)**		
Finnmark (N)	0.39 (0.23 - 0.66)	0.55 (0.43 - 0.70)	North	0.60 (0.51 - 0.71)	0.76 (0.70 - 0.83)
UNN (N)	0.49 (0.37 - 0.65)	0.73 (0.63 - 0.85)	Central	1.42 (1.27 - 1.58)	1.47 (1.39 - 1.57)
Nordland (N)	0.43 (0.31 - 0.61)	0.72 (0.62 - 0.85)	West	0.80 (0.72 - 0.88)	1.01 (0.95 - 1.07)
Helgeland (N)	0.70 (0.51 - 0.96)	0.68 (0.56 - 0.82)	South-East	1.0 (ref)	1.0 (ref)
Nord-Trøndelag (C)	0.82 (0.63 - 1.06)	1.02 (0.87 - 1.18)			
St. Olavs (C)	1.47 (1.24 - 1.75)	1.69 (1.53 - 1.86)			
Møre-Romsdal (C)	1.15 (0.95 - 1.39)	1.14 (1.02 - 1.28)			
Førde (W)	0.78 (0.58 - 1.06)	0.76 (0.63 - 0.91)			
Bergen (W)	1.06 (0.90 - 1.26)	1.22 (1.11 - 1.35)			
Fonna (W)	1.14 (0.92 - 1.41)	1.20 (1.06 - 1.36)			
Stavanger (W)	0.38 (0.32 - 0.45)	0.64 (0.57 - 0.71)			
Østfold (SE)	0.78 (0.64 - 0.95)	0.86 (0.77 - 0.97)			
Akershus (SE)	0.81 (0.68 - 0.95)	0.90 (0.81 - 1.00)			
OUS (SE)	0.92 (0.75 - 1.13)	1.03 (0.91 - 1.16)			
Lovisenberg (SE)	0.96 (0.71 - 1.32)	0.87 (0.71 - 1.06)			
Diakonhjemmet (SE)	1.01 (0.78 - 1.30)	1.17 (1.01 - 1.35)			
Innlandet (SE)	0.64 (0.53 - 0.77)	0.71 (0.63 - 0.79)			
Vestre Viken (SE)	1.0 (ref)	1.0 (ref)			
Vestfold (SE)	0.75 (0.62 - 0.91)	0.83 (0.73 - 0.94)			
Telemark (SE)	1.00 (0.82 - 1.23)	1.04 (0.92 - 1.18)			
Sørlandet (SE)	0.86 (0.71 - 1.04)	0.92 (0.82 - 1.03)			

B: 2008-2010 - Before capacity increase

Table B1: Characteristics of patients 2008-2010.

	Atrial fibrillation			Ablation (% proportion with ablation)		
	Female	Male	Total	Female	Male	Total
Number of patients	7 844	17 949	25 793	287 (3.7%)	1 139 (6.3%)	1 426 (5.5%)
**Age group‡**						
Under 50	456	1 538	1 994	49 (10.7%)	230 (15.0%)	279 (14.0%)
50-59	966	3 445	4 411	86 (8.9%)	364 (10.6%)	450 (10.2%)
60-69	3 307	7 873	11 180	123 (3.7%)	441 (5.6%)	564 (5.0%)
70 and older	3 115	5 093	8 208	29 (0.9%)	104 (2.0%)	133 (1.6%)
**Education‡**						
Low	2 647	4 128	6 775	68 (2.6%)	155 (3.8%)	223 (3.3%)
Medium	3 603	8 736	12 339	123 (3.4%)	537 (6.1%)	660 (5.3%)
High	1 594	5 085	6 679	96 (6.0%)	447 (8.8%)	543 (8.1%)
**Income‡**						
Low	4 761	4 145	8 906	126 (2.6%)	134 (3.2%)	260 (2.9%)
Medium	2 611	8 842	11 453	131 (5.0%)	508 (5.7%)	639 (5.6%)
High	472	4 962	5 434	30 (6.4%)	497 (10.0%)	527 (9.7%)
**Hospital referral area‡** ∗						
Finnmark (N)	85	257	342	1 (1.2%)	9 (3.5%)	10 (2.9%)
UNN (N)	310	740	1 050	10 (3.2%)	41 (5.5%)	51 (4.9%)
Nordland (N)	225	574	799	6 (2.7%)	35 (6.1%)	41 (5.1%)
Helgeland (N)	148	346	494	10 (6.8%)	17 (4.9%)	27 (5.5%)
Nord-Trøndelag (C)	191	458	649	9 (4.7%)	30 (6.6%)	39 (6.0%)
St. Olavs (C)	400	1 051	1 451	16 (4.0%)	112 (10.7%)	128 (8.8%)
Møre-Romsdal (C)	342	861	1 203	18 (5.3%)	71 (8.2%)	89 (7.4%)
Førde (W)	165	424	589	7 (4.2%)	20 (4.7%)	27 (4.6%)
Bergen (W)	532	1 355	1 887	31 (5.8%)	122 (9.0%)	153 (8.1%)
Fonna (W)	251	648	899	11 (4.4%)	42 (6.5%)	53 (5.9%)
Stavanger (W)	500	1 134	1 634	23 (4.6%)	80 (7.1%)	103 (6.3%)
Østfold (SE)	445	985	1 430	12 (2.7%)	50 (5.1%)	62 (4.3%)
Akershus (SE)	814	1 631	2 445	27 (3.3%)	92 (5.6%)	119 (4.9%)
OUS (SE)	362	814	1 176	14 (3.9%)	56 (6.9%)	70 (6.0%)
Lovisenberg (SE)	95	212	307	5 (5.3%)	13 (6.1%)	18 (5.9%)
Diakonhjemmet (SE)	179	435	614	6 (3.4%)	26 (6.0%)	32 (5.2%)
Innlandet (SE)	666	1 463	2 129	13 (2.0%)	60 (4.1%)	73 (3.4%)
Vestre Viken (SE)	869	1 838	2 707	40 (4.6%)	137 (7.5%)	177 (6.5%)
Vestfold (SE)	481	979	1 460	14 (2.9%)	41 (4.2%)	55 (3.8%)
Telemark (SE)	323	746	1 069	8 (2.5%)	40 (5.4%)	48 (4.5%)
Sørlandet (SE)	461	998	1 459	6 (1.3%)	45 (4.5%)	51 (3.5%)

† Event is ablation, death, emigration or end of study period. For ablation patients the event is ablation.
‡ At the time of diagnosis.
∗ The four regional health authorities: N - North, C - Central, W - West and SE - South-East.

Table B2: Multivariable Cox regression, separate by gender, 2008-2010. Hazard ratios (95% confidence interval), adjusted for follow-up time.

	Model 1 (HT)			Model 2 (RHA)	
	Female	Male		Female	Male
**Education**			**Education**		
Low	1.0 (ref)	1.0 (ref)	Low	1.0 (ref)	1.0 (ref)
Medium	1.13 (0.83 - 1.53)	1.33 (1.11 - 1.60)	Medium	1.12 (0.83 - 1.52)	1.34 (1.11 - 1.60)
High	1.45 (1.02 - 2.06)	1.62 (1.33 - 1.97)	High	1.48 (1.04 - 2.10)	1.65 (1.36 - 2.01)
**Income**			**Income**		
Low	1.0 (ref)	1.0 (ref)	Low	1.0 (ref)	1.0 (ref)
Medium	1.11 (0.84 - 1.46)	1.43 (1.18 - 1.74)	Medium	1.14 (0.86 - 1.49)	1.44 (1.19 - 1.75)
High	1.06 (0.68 - 1.65)	1.77 (1.44 - 2.18)	High	1.12 (0.72 - 1.74)	1.81 (1.48 - 2.23)
**Follow-up time (years)**			**Follow-up time (years)**		
1	1.0 (ref)	1.0 (ref)	1	1.0 (ref)	1.0 (ref)
2	0.95 (0.73 - 1.22)	0.93 (0.82 - 1.06)	2	0.94 (0.73 - 1.21)	0.93 (0.82 - 1.05)
3	0.55 (0.37 - 0.81)	0.61 (0.50 - 0.74)	3	0.54 (0.37 - 0.81)	0.61 (0.50 - 0.73)
**Hospital referral area (HT)**			**Regional referral area (RHA)**		
Finnmark (N)	0.22 (0.03 - 1.62)	0.57 (0.29 - 1.13)	North	1.22 (0.81 - 1.85)	1.07 (0.87 - 1.32)
UNN (N)	0.86 (0.43 - 1.73)	0.81 (0.57 - 1.15)	Central	1.49 (1.06 - 2.10)	1.79 (1.53 - 2.10)
Nordland (N)	0.67 (0.28 - 1.59)	1.00 (0.69 - 1.46)	West	1.51 (1.13 - 2.01)	1.43 (1.24 - 1.66)
Helgeland (N)	1.54 (0.76 - 3.10)	0.77 (0.47 - 1.29)	South-East	1.0 (ref)	1.0 (ref)
Nord-Trøndelag (C)	1.02 (0.49 - 2.12)	1.11 (0.75 - 1.65)			
St. Olavs (C)	0.93 (0.52 - 1.66)	1.54 (1.20 - 1.97)			
Møre-Romsdal (C)	1.17 (0.67 - 2.05)	1.33 (1.00 - 1.78)			
Førde (W)	1.05 (0.47 - 2.35)	0.79 (0.49 - 1.27)			
Bergen (W)	1.40 (0.88 - 2.25)	1.38 (1.08 - 1.76)			
Fonna (W)	0.96 (0.49 - 1.89)	0.99 (0.70 - 1.40)			
Stavanger (W)	0.81 (0.48 - 1.36)	0.98 (0.74 - 1.30)			
Østfold (SE)	0.64 (0.33 - 1.22)	0.72 (0.52 - 1.00)			
Akershus (SE)	0.75 (0.46 - 1.23)	0.80 (0.61 - 1.04)			
OUS (SE)	0.91 (0.49 - 1.67)	0.85 (0.62 - 1.16)			
Lovisenberg (SE)	1.07 (0.42 - 2.73)	0.77 (0.44 - 1.37)			
Diakonhjemmet (SE)	0.67 (0.28 - 1.58)	0.69 (0.45 - 1.05)			
Innlandet (SE)	0.47 (0.25 - 0.88)	0.67 (0.50 - 0.92)			
Vestre Viken (SE)	1.0 (ref)	1.0 (ref)			
Vestfold (SE)	0.66 (0.36 - 1.21)	0.59 (0.42 - 0.84)			
Telemark (SE)	0.57 (0.27 - 1.22)	0.75 (0.53 - 1.07)			
Sørlandet (SE)	0.29 (0.12 - 0.69)	0.68 (0.48 - 0.95)			

C: 2011-2017 - After capacity increase

Table C1: Characteristics of patients 2011-2017.

	Atrial fibrillation			Ablation (% proportion with ablation)		
	Female	Male	Total	Female	Male	Total
Number of patients	26 887	55 415	82 302	2 415 (9.0%)	6 935 (12.5%)	9 350 (11.4%)
**Age group‡**						
Under 50	2 372	5 411	7 783	392 (16.5%)	1 217 (22.5%)	1 609 (20.7%)
50-59	3 516	9 612	13 128	514 (14.6%)	2 052 (21.3%)	2 566 (19.5%)
60-69	10 386	23 003	33 389	1 095 (10.5%)	2 845 (12.4%)	3 940 (11.8%)
70 and older	10 613	17 389	28 002	414 (3.9%)	821 (4.7%)	1 235 (4.4%)
**Education‡**						
Low	7 668	12 152	19 820	494 (6.4%)	996 (8.2%)	1 490 (7.5%)
Medium	12 608	27 277	39 885	1 151 (9.1%)	3 223 (11.8%)	4 374 (11.0%)
High	6 611	15 986	22 597	770 (11.6%)	2 716 (17.0%)	3 486 (15.4%)
**Income‡**						
Low	12 454	8 909	21 363	845 (6.8%)	620 (7.0%)	1 465 (6.9%)
Medium	11 587	27 607	39 194	1 191 (10.3%)	2 986 (10.8%)	4 177 (10.7%)
High	2 846	18 899	21 745	379 (13.3%)	3 329 (17.6%)	3 708 (17.1%)
**Hospital referral area‡** ∗						
Finnmark (N)	298	795	1 093	14 (4.7%)	59 (7.4%)	73 (6.7%)
UNN (N)	877	2 032	2 909	46 (5.2%)	205 (10.1%)	251 (8.6%)
Nordland (N)	688	1 595	2 283	29 (4.2%)	147 (9.2%)	176 (7.7%)
Helgeland (N)	478	1 015	1 493	33 (6.9%)	97 (9.6%)	130 (8.7%)
Nord-Trøndelag (C)	677	1 468	2 145	53 (7.8%)	191 (13.0%)	244 (11.4%)
St. Olavs (C)	1 260	2 962	4 222	192 (15.2%)	627 (21.2%)	819 (19.4%)
Møre-Romsdal (C)	1 151	2 585	3 736	141 (12.3%)	372 (14.4%)	513 (13.7%)
Førde (W)	525	1 240	1 765	37 (7.0%)	122 (9.8%)	159 (9.0%)
Bergen (W)	1 806	4 184	5 990	183 (10.1%)	624 (14.9%)	807 (13.5%)
Fonna (W)	856	1 958	2 814	103 (12.0%)	281 (14.4%)	384 (13.6%)
Stavanger (W)	3 173	4 920	8 093	160 (5.0%)	445 (9.0%)	605 (7.5%)
Østfold (SE)	1 557	3 165	4 722	126 (8.1%)	370 (11.7%)	496 (10.5%)
Akershus (SE)	2 568	4 897	7 465	219 (8.5%)	596 (12.2%)	815 (10.9%)
OUS (SE)	1 136	2 434	3 570	119 (10.5%)	344 (14.1%)	463 (13.0%)
Lovisenberg (SE)	342	739	1 081	41 (12.0%)	97 (13.1%)	138 (12.8%)
Diakonhjemmet (SE)	559	1 289	1 848	59 (10.6%)	203 (15.7%)	262 (14.2%)
Innlandet (SE)	2 296	4 638	6 934	161 (7.0%)	428 (9.2%)	589 (8.5%)
Vestre Viken (SE)	2 627	5 316	7 943	297 (11.3%)	705 (13.3%)	1 002 (12.6%)
Vestfold (SE)	1 421	2 792	4 213	127 (8.9%)	320 (11.5%)	447 (10.6%)
Telemark (SE)	1 019	2 156	3 175	118 (11.6%)	309 (14.3%)	427 (13.4%)
Sørlandet (SE)	1 573	3 235	4 808	157 (10.0%)	393 (12.1%)	550 (11.4%)

† Event is ablation, death, emigration or end of study period. For ablation patients the event is ablation.
‡ At the time of diagnosis.
∗ The four regional health authorities: N - North, C - Central, W - West and SE - South-East.

Table C2: Multivariable Cox regression, separate by gender, 2011-2017. Hazard ratios (95% confidence interval), adjusted for follow-up time.

	Model 1 (HT)			Model 2 (RHA)	
	Female	Male		Female	Male
**Education**			**Education**		
Low	1.0 (ref)	1.0 (ref)	Low	1.0 (ref)	1.0 (ref)
Medium	1.27 (1.14 - 1.41)	1.29 (1.20 - 1.39)	Medium	1.28 (1.15 - 1.42)	1.31 (1.21 - 1.40)
High	1.29 (1.14 - 1.47)	1.66 (1.54 - 1.80)	High	1.32 (1.17 - 1.49)	1.71 (1.58 - 1.84)
**Income**			**Income**		
Low	1.0 (ref)	1.0 (ref)	Low	1.0 (ref)	1.0 (ref)
Medium	1.21 (1.10 - 1.33)	1.49 (1.36 - 1.62)	Medium	1.21 (1.10 - 1.33)	1.49 (1.36 - 1.62)
High	1.34 (1.17 - 1.54)	1.71 (1.57 - 1.88)	High	1.32 (1.15 - 1.51)	1.70 (1.55 - 1.86)
**Follow-up time (years)**			**Follow-up time (years)**		
1	1.0 (ref)	1.0 (ref)	1	1.0 (ref)	1.0 (ref)
2	0.58 (0.53 - 0.65)	0.69 (0.65 - 0.73)	2	0.58 (0.52 - 0.64)	0.68 (0.65 - 0.73)
3	0.46 (0.40 - 0.52)	0.45 (0.42 - 0.49)	3	0.45 (0.40 - 0.51)	0.45 (0.42 - 0.49)
4	0.40 (0.34 - 0.47)	0.41 (0.37 - 0.45)	4	0.39 (0.34 - 0.46)	0.41 (0.37 - 0.45)
5	0.52 (0.44 - 0.62)	0.39 (0.35 - 0.43)	5	0.52 (0.44 - 0.61)	0.38 (0.34 - 0.43)
6	0.50 (0.40 - 0.62)	0.46 (0.41 - 0.53)	6	0.50 (0.40 - 0.62)	0.46 (0.41 - 0.53)
7	0.35 (0.24 - 0.50)	0.34 (0.27 - 0.42)	7	0.35 (0.24 - 0.50)	0.34 (0.27 - 0.42)
**Hospital referral area (HT)**			**Regional referral area (RHA)**		
Finnmark (N)	0.42 (0.24 - 0.72)	0.61 (0.47 - 0.79)	North	0.55 (0.46 - 0.66)	0.80 (0.73 - 0.87)
UNN (N)	0.49 (0.36 - 0.66)	0.83 (0.71 - 0.97)	Central	1.40 (1.25 - 1.57)	1.52 (1.42 - 1.62)
Nordland (N)	0.41 (0.28 - 0.60)	0.78 (0.65 - 0.93)	West	0.69 (0.62 - 0.77)	0.93 (0.87 - 0.99)
Helgeland (N)	0.58 (0.40 - 0.83)	0.80 (0.65 - 0.99)	South-East	1.0 (ref)	1.0 (ref)
Nord-Trøndelag (C)	0.74 (0.56 - 1.00)	1.14 (0.97 - 1.34)			
St. Olavs (C)	1.55 (1.29 - 1.86)	1.86 (1.67 - 2.07)			
Møre-Romsdal (C)	1.17 (0.96 - 1.43)	1.24 (1.09 - 1.41)			
Førde (W)	0.68 (0.48 - 0.96)	0.84 (0.69 - 1.02)			
Bergen (W)	0.98 (0.81 - 1.17)	1.21 (1.08 - 1.34)			
Fonna (W)	1.20 (0.96 - 1.50)	1.27 (1.10 - 1.46)			
Stavanger (W)	0.31 (0.26 - 0.38)	0.60 (0.53 - 0.67)			
Østfold (SE)	0.81 (0.66 - 1.00)	0.99 (0.87 - 1.13)			
Akershus (SE)	0.78 (0.65 - 0.93)	0.98 (0.87 - 1.09)			
OUS (SE)	0.92 (0.74 - 1.14)	1.01 (0.89 - 1.15)			
Lovisenberg (SE)	0.98 (0.71 - 1.36)	0.92 (0.75 - 1.14)			
Diakonhjemmet (SE)	0.91 (0.68 - 1.20)	1.06 (0.91 - 1.25)			
Innlandet (SE)	0.67 (0.56 - 0.82)	0.80 (0.71 - 0.91)			
Vestre Viken (SE)	1.0 (ref)	1.0 (ref)			
Vestfold (SE)	0.79 (0.64 - 0.98)	0.91 (0.80 - 1.04)			
Telemark (SE)	1.12 (0.90 - 1.38)	1.19 (1.04 - 1.36)			
Sørlandet (SE)	0.95 (0.79 - 1.16)	1.00 (0.88 - 1.13)			

D: 2013-2017 - Fibrillation only

Table D1: Characteristics of patients 2013-2017.

	Atrial fibrillation			Ablation (% proportion with ablation)		
	Female	Male	Total	Female	Male	Total
Number of patients	19 619	42 341	61 960	1 543 (7.9%)	4 604 (10.9%)	6 147 (9.9%)
**Age group‡**						
Under 50	1 046	3 548	4 594	157 (15.0%)	697 (19.6%)	854 (18.6%)
50-59	2 373	6 930	9 303	309 (13.0%)	1 260 (18.2%)	1 569 (16.9%)
60-69	7 675	17 460	25 135	724 (9.4%)	1 983 (11.4%)	2 707 (10.8%)
70 and older	8 525	14 403	22 928	353 (4.1%)	664 (4.6%)	1 017 (4.4%)
**Education‡**						
Low	5 491	9 002	14 493	326 (5.9%)	630 (7.0%)	956 (6.6%)
Medium	9 338	20 816	30 154	743 (8.0%)	2 144 (10.3%)	2 887 (9.6%)
High	4 790	12 523	17 313	474 (9.9%)	1 830 (14.6%)	2 304 (13.3%)
**Income‡**						
Low	8 885	6 343	15 228	544 (6.1%)	365 (5.8%)	909 (6.0%)
Medium	8 642	21 178	29 820	746 (8.6%)	1 928 (9.1%)	2 674 (9.0%)
High	2 092	14 820	16 912	253 (12.1%)	2 311 (15.6%)	2 564 (15.2%)
**Hospital referral area‡** ∗						
Finnmark (N)	229	616	845	7 (3.1%)	42 (6.8%)	49 (5.8%)
UNN (N)	728	1 664	2 392	31 (4.3%)	136 (8.2%)	167 (7.0%)
Nordland (N)	575	1 310	1 885	21 (3.7%)	105 (8.0%)	126 (6.7%)
Helgeland (N)	385	793	1 178	23 (6.0%)	62 (7.8%)	85 (7.2%)
Nord-Trøndelag (C)	516	1 133	1 649	41 (7.9%)	142 (12.5%)	183 (11.1%)
St. Olavs (C)	941	2 269	3 210	153 (16.3%)	457 (20.1%)	610 (19.0%)
Møre-Romsdal (C)	883	1 998	2 881	110 (12.5%)	277 (13.9%)	387 (13.4%)
Førde (W)	432	1 004	1 436	28 (6.5%)	87 (8.7%)	115 (8.0%)
Bergen (W)	1 471	3 375	4 846	133 (9.0%)	434 (12.9%)	567 (11.7%)
Fonna (W)	655	1 457	2 112	75 (11.5%)	183 (12.6%)	258 (12.2%)
Stavanger (W)	1 259	2 693	3 952	81 (6.4%)	260 (9.7%)	341 (8.6%)
Østfold (SE)	1 115	2 310	3 425	85 (7.6%)	239 (10.3%)	324 (9.5%)
Akershus (SE)	2 025	3 856	5 881	141 (7.0%)	372 (9.6%)	513 (8.7%)
OUS (SE)	862	1 899	2 761	73 (8.5%)	225 (11.8%)	298 (10.8%)
Lovisenberg (SE)	286	646	932	22 (7.7%)	67 (10.4%)	89 (9.5%)
Diakonhjemmet (SE)	418	1 014	1 432	31 (7.4%)	148 (14.6%)	179 (12.5%)
Innlandet (SE)	1 800	3 660	5 460	98 (5.4%)	270 (7.4%)	368 (6.7%)
Vestre Viken (SE)	2 009	4 189	6 198	154 (7.7%)	421 (10.1%)	575 (9.3%)
Vestfold (SE)	1 026	2 103	3 129	75 (7.3%)	198 (9.4%)	273 (8.7%)
Telemark (SE)	733	1 655	2 388	65 (8.9%)	207 (12.5%)	272 (11.4%)
Sørlandet (SE)	1 271	2 697	3 968	96 (7.6%)	272 (10.1%)	368 (9.3%)

† Event is ablation, death, emigration or end of study period. For ablation patients the event is ablation.
‡ At the time of diagnosis.
∗ The four regional health authorities: N - North, C - Central, W - West and SE - South-East.

Table D2: Multivariable Cox regression, separate by gender, 2013-2017. Hazard ratios (95% confidence interval), adjusted for follow-up time.

	Model 1 (HT)			Model 2 (RHA)	
	Female	Male		Female	Male
**Education**			**Education**		
Low	1.0 (ref)	1.0 (ref)	Low	1.0 (ref)	1.0 (ref)
Medium	1.22 (1.07 - 1.40)	1.30 (1.19 - 1.42)	Medium	1.23 (1.08 - 1.41)	1.31 (1.20 - 1.43)
High	1.25 (1.07 - 1.46)	1.66 (1.51 - 1.83)	High	1.27 (1.08 - 1.48)	1.70 (1.54 - 1.87)
**Income**			**Income**		
Low	1.0 (ref)	1.0 (ref)	Low	1.0 (ref)	1.0 (ref)
Medium	1.16 (1.03 - 1.30)	1.52 (1.36 - 1.71)	Medium	1.16 (1.03 - 1.30)	1.53 (1.37 - 1.71)
High	1.35 (1.13 - 1.60)	1.81 (1.61 - 2.03)	High	1.33 (1.12 - 1.58)	1.82 (1.62 - 2.05)
**Follow-up time (years)**			**Follow-up time (years)**		
1	1.0 (ref)	1.0 (ref)	1	1.0 (ref)	1.0 (ref)
2	0.63 (0.55 - 0.71)	0.63 (0.59 - 0.68)	2	0.62 (0.55 - 0.71)	0.63 (0.59 - 0.68)
3	0.48 (0.41 - 0.56)	0.42 (0.38 - 0.46)	3	0.47 (0.41 - 0.55)	0.41 (0.38 - 0.46)
4	0.39 (0.32 - 0.47)	0.35 (0.31 - 0.39)	4	0.38 (0.31 - 0.47)	0.34 (0.31 - 0.39)
5	0.21 (0.14 - 0.30)	0.21 (0.17 - 0.26)	5	0.21 (0.14 - 0.30)	0.21 (0.17 - 0.26)
**Hospital referral area (HT)**			**Regional referral area (RHA)**		
Finnmark (N)	0.39 (0.18 - 0.83)	0.76 (0.55 - 1.04)	North	0.59 (0.47 - 0.73)	0.83 (0.74 - 0.93)
UNN (N)	0.59 (0.40 - 0.86)	0.89 (0.73 - 1.07)	Central	1.87 (1.64 - 2.14)	1.77 (1.64 - 1.91)
Nordland (N)	0.51 (0.32 - 0.81)	0.89 (0.72 - 1.11)	West	1.12 (0.98 - 1.27)	1.12 (1.04 - 1.20)
Helgeland (N)	0.71 (0.46 - 1.10)	0.85 (0.65 - 1.11)	South-East	1.0 (ref)	1.0 (ref)
Nord-Trøndelag (C)	1.08 (0.77 - 1.53)	1.41 (1.17 - 1.71)			
St. Olavs (C)	2.37 (1.89 - 2.96)	2.30 (2.02 - 2.63)			
Møre-Romsdal (C)	1.72 (1.34 - 2.19)	1.57 (1.35 - 1.83)			
Førde (W)	0.91 (0.61 - 1.36)	0.96 (0.76 - 1.21)			
Bergen (W)	1.24 (0.99 - 1.57)	1.32 (1.16 - 1.51)			
Fonna (W)	1.69 (1.28 - 2.23)	1.42 (1.19 - 1.69)			
Stavanger (W)	0.74 (0.56 - 0.96)	0.93 (0.80 - 1.08)			
Østfold (SE)	1.09 (0.84 - 1.42)	1.12 (0.96 - 1.32)			
Akershus (SE)	0.91 (0.72 - 1.14)	0.99 (0.87 - 1.14)			
OUS (SE)	1.10 (0.83 - 1.45)	1.15 (0.98 - 1.36)			
Lovisenberg (SE)	0.91 (0.58 - 1.43)	0.94 (0.72 - 1.22)			
Diakonhjemmet (SE)	0.93 (0.63 - 1.37)	1.33 (1.10 - 1.60)			
Innlandet (SE)	0.75 (0.58 - 0.96)	0.84 (0.72 - 0.98)			
Vestre Viken (SE)	1.0 (ref)	1.0 (ref)			
Vestfold (SE)	1.00 (0.76 - 1.31)	1.00 (0.84 - 1.18)			
Telemark (SE)	1.23 (0.92 - 1.64)	1.35 (1.14 - 1.59)			
Sørlandet (SE)	1.05 (0.81 - 1.35)	1.06 (0.91 - 1.24)			
